# Improved Draft Genome Sequence of *Microcystis aeruginosa* NIES-298, a Microcystin-Producing Cyanobacterium from Lake Kasumigaura, Japan

**DOI:** 10.1128/genomeA.01551-17

**Published:** 2018-02-01

**Authors:** Haruyo Yamaguchi, Shigekatsu Suzuki, Masanobu Kawachi

**Affiliations:** a Center for Environmental Biology and Ecosystem Studies, National Institute for Environmental Studies, Ibaraki, Japan

## Abstract

*Microcystis aeruginosa* is a globally well-known bloom-forming cyanobacterium. An improved draft whole-genome sequence of *M. aeruginosa* NIES-298, which is a microcystin-producing strain isolated from Lake Kasumigaura, Japan, is published here. The genome comprises approximately 5.0 Mbp, with an average G+C content of 42.6% and 4,537 predicted protein-coding genes.

## GENOME ANNOUNCEMENT

*Microcystis aeruginosa* is a problematic cyanobacterium because it forms massive blooms and produces bad odors in freshwater environments (1). The most serious problem associated with this species is the production of cyanotoxins (microcystins) (1). It is known that *M. aeruginosa* is genetically divided into at least eight clades (groups A to G and X) based on a multilocus phylogenetic analysis (2). Of the clades, A, X, and a part of B produce microcystins. *M. aeruginosa* NIES-298 is a microcystin-producing strain isolated from Lake Kasumigaura, Japan (3). The strain is available from the Microbial Culture Collection at the National Institute for Environmental Studies, Japan (http://mcc.nies.go.jp/). According to phylogenetic analysis, this strain is assigned to group B (2). We have reported the genome sequence of *M. aeruginosa* NIES-98, which is a non-microcystin-producing strain from Lake Kasumigaura assigned to group B (4). Although Rhee et al. published a draft genome sequence of NIES-298 (5), the quality of the genome sequence was insufficient. To improve this, we performed genome sequencing of NIES-298 using paired-end and mate-pair reads generated by an Illumina MiSeq platform. This information provides a better understanding of the evolutionary history of group B in *M. aeruginosa*.

Extraction of genomic DNA was performed with 10 ml of the axenic culture of NIES-298 using the DNeasy Plant minikit (Qiagen), which was then fragmented using a Covaris M220 focused-ultrasonicator (Covaris) to approximately 550 bp. Genomic libraries of paired-end and mate-pair reads were constructed using the TruSeq Nano DNA library prep kit (Illumina) and the Nextera mate-pair library preparation kit (Illumina), respectively. Next-generation sequencing was performed via the MiSeq platform (Illumina) using the 600-cycle MiSeq reagent kit version 3. The resultant paired-end and mate-pair reads were 551,378,644 bp and 115,840,891 bp, respectively. Low-quality reads/bases were filtered using Trimmomatic version 0.36 (6), and a *de novo* assembly was performed by SPAdes 3.7.1 (7). The resulting genome comprised 97 contigs of 4,957,478 bp. The average genome coverage of the paired-end reads was 111.2×. The maximum length of a contig was 556,706 bp, and the mean contig size was 51,108 bp. The draft genome of NIES-298 was annotated with the DDBJ Fast Annotation and Submission Tool (DFAST) (8). The genome comprised 4,537 predicted protein-coding sequences (CDSs), one set of rRNA, and 48 tRNA genes. The G+C content of the genome was 42.6%. antiSMASH (9) predicted 34 secondary metabolite gene clusters, including microcystin, aeruginosin, microviridin, microcyclamide, and micropeptin biosynthesis gene clusters in the genome. The sequence of 16S rRNA was compared with that of NIES-98, resulting in 96.4% similarity. OrthoFinder (10) identified 3,488 orthologous gene groups between the 2 genomes. Additional genome sequences would help in understanding the complex evolution of *M. aeruginosa*.

### Accession number(s).

This whole-genome shotgun project has been deposited in GenBank under the accession no. BEYQ01000001 to BEYQ01000097.
